# ^18^F-Florbetaben PET Detection of Cardiac and Multi-Organ Involvement for Identifying AL and ATTR Cardiac Amyloidosis

**DOI:** 10.1016/j.jacadv.2026.102626

**Published:** 2026-03-25

**Authors:** Telma Sprauel, Emmanuel Itti, Laetitia Imbert, Timothée Zaragori, Matthieu Doyen, Véronique Roch, Antoine Verger, Olivier Huttin, Caroline Jacquet, Erwan Donal, Florence Lejeune, Nicolas Veran, Juliette Piccoli, Olivier Lairez, Anne Hitzel, Eve Cariou, Benjamin Chambert, Camille Soullier, Amira Zaroui, Gabriela Hossu, Thibaud Damy, Pierre-Yves Marie

**Affiliations:** a Department of Nuclear Medicine, CHRU Nancy, and Nancyclotep Platform, France; b Department of Nuclear Medicine, CHU Henri-Mondor, Créteil, France; c Université de Lorraine, IADI, INSERM U1254, Nancy, France; d CHRU-Nancy, Inserm, Université de Lorraine, Nancy, France; e Department of Cardiology, CHRU Nancy, Nancy, France; f Université de Lorraine, DCAC, INSERM U1254, Nancy, France; g Department of Haematology, CHRU Nancy, Nancy, France; h Department of Cardiology, CHU Rennes, Rennes, France; i Université de Rennes, INSERM U1099, Rennes, France; j Department of Nuclear Medicine, Centre Eugène Marquis, Rennes, France; k Department of Cardiac Surgery, CHRU Nancy, Nancy, France; l Department of Nuclear Medicine, CHU Toulouse, Toulouse, France; m University of Toulouse, Toulouse University Hospital, HealthAge Institute, Toulouse, France; n Department of Cardiology, CHU Toulouse, Toulouse, France; o Department of Nuclear Medicine, CHU Nimes, Nimes, France; p Department of Cardiology, CHU Nimes, Nimes, France; q Refferal Center for Cardiac Amyloidosis and Réseau Amylose, CHU Henri-Mondor, Créteil, France; r Department of Cardiology, CHU Henri-Mondor and Université Paris Est Créteil, Créteil, France

**Keywords:** cardiac amyloidosis, ^18^F-florbetaben, PET, whole-body

## Abstract

**Background:**

^18^F-florbetaben positron emission tomography (PET) is reported to detect i) cardiac amyloidosis (CA), particularly AL (light chain) forms, and ii) sites of extracardiac AL-amyloid infiltrates.

**Objectives:**

The purpose of this study was to identify CA and differentiate AL-CA from ATTR-CA (transthyretin) by evaluating cardiac and multi-organ involvement with whole-body ^18^F-florbetaben PET.

**Methods:**

Multicentric study of 61 patients with left ventricular hypertrophy due to AL-CA (n = 25), ATTR-CA (n = 25), and aortic stenosis (n = 11, controls). A 20-minute whole-body PET was preceded by a 10-minute dynamic cardiac PET recording started during ^18^F-florbetaben injection.

**Results:**

Tracer uptake was significantly increased in whole-body PET of CA patients compared to controls, with AL-CA patients, in particular, exhibiting increased uptake in myocardium, lung, and spleen, and decreased uptake in blood, salivary glands, skeletal muscle, and liver. Among cardiac parameters, the myocardial/blood standardized uptake value (SUV)max ratio from whole-body PET best differentiated AL-CA from ATTR-CA, with 90% (55/61) of patients identified correctly (kappa value: 0.819) using SUVmax ratio thresholds of 2-to-4 for ATTR-CA and >4 for AL-CA. When combining cardiac with extracardiac PET variables, the selected multivariate predictors were myocardial uptake volume and the lung and salivary gland SUVmean. Thresholds of myocardial uptake volume >10 mL for CA and lung/salivary gland SUVmean >0.285 for AL-CA correctly identified 93% (57/61) of patients (kappa value: 0.871). PET variables improved the overall prediction provided by the bone scan Perugini score (*P* < 0.001).

**Conclusions:**

Whole-body ^18^F-florbetaben PET identifies CA and differentiates AL-CA from ATTR-CA. These distinctions are strengthened by combining assessments of cardiac and multi-organ involvement.

Recent advances in treatment have established the benefits of early diagnosis in cardiac amyloidosis (CA) patients, thereby improving prognosis.^1^ However, a noninvasive diagnosis of CA remains challenging, with bone scintigraphy mostly detecting transthyretin amyloidosis (ATTR) type, and magnetic resonance imaging (MRI) not specific or efficient enough for differentiating ATTR from immunoglobulin light chain amyloidosis (AL).^2^ Several amyloid positron emission tomography (PET) tracers, such as [^18^F]-florbetaben, nonetheless, provide favorable properties for detecting CA, particularly AL forms and when cardiac kinetic parameters are analyzed.^3^, ^4^, ^5^

[^18^F]-florbetaben PET may additionally detect histologically confirmed AL-amyloid infiltrates in specific extracardiac tissues (lung, liver, and thyroid).^6^^,^^7^ This raises the prospect of whole-body PET as a means of comprehensively detecting the majority of cardiac and extracardiac amyloid deposition sites and thereby identifying CA as well as differentiating AL from ATTR types. Albeit that optimal recording times for whole-body [^18^F]-florbetaben PET, are constrained by i) excessive blood activities in the minutes following tracer injection and ii) the high clearance rate of [^18^F]-florbetaben from ATTR-CA areas.

Based on previously published [^18^F]-florbetaben pharmacokinetic studies^3^^,^^4^ and confirmed from results of 2 ATTR-CA patients (see Supplemental Figure 1), we hypothesize that a whole-body PET recording, starting at the 10th minute post tracer injection and ending at the 30th minute, provides an accurate assessment of amyloid infiltration sites on the whole-body scale of both AL- and ATTR-amyloidosis patients.

This current prospective multicentric CAPRI (Cardiac Amyloidosis PET Radionuclide Imaging) study assesses the ability of whole-body [^18^F]-florbetaben PET to differentiate AL from ATTR-CA patients, compared to a control population presenting with aortic stenosis–related left ventricular (LV) hypertrophy, and when considering the multi-organ involvement commonly seen in AL-CA.

## Methods

### Patient selection and study design

Eligible patients were assigned to 3 groups—ATTR-CA, AL-CA, and controls—in 5 French university hospitals in Nancy, Creteil, Rennes, Toulouse, and Nimes. The main inclusion criteria comprised: 1) for ATTR-CA, a positive biopsy or a positive bone scan (≥2 Perugini score on planar images recorded around 3 hours after injection of [^99m^Tc]-3,3-diphosphono-1,2-propanedicarboxylic acid [DPD] or -hydroxymethylene diphosphonate [HMDP]),^8^ with no family history of the disease, suspected wild-type ATTR; 2) for AL-CA, a positive biopsy associated with an abnormal NT-proBNP, BNP, or troponin-I, plasma level; and 3) for controls, a recent history of surgical or transcatheter aortic valve implantation aortic stenosis treatment. In all biopsy cases, amyloid should be identified by examining biopsy specimens under polarized light after Congo red staining, and subtyping should be achieved by immunohistochemistry.

All study patients also had an increase in LV diastolic septal thickness (≥13 mm by echocardiography), no apparent cause of cardiac disease (other than CA in the CA groups and aortic stenosis in the control group), LV function with an ejection fraction >35%, and no severe hepatic or renal failure.

After giving their written informed consent, patients were referred for [^18^F]-florbetaben PET. This was preceded by bone scintigraphy and blood sampling for plasma analysis with light-chain dosages, if these 2 tests had not already been performed in the last 3 months, if they were conducted incorrectly, or if results from these 2 tests could not be retrieved for reanalysis by the Nancy Core Laboratory. We further excluded ATTR-CA and control patients with a monoclonal gammopathy associated with an abnormal free light-chain ratio (<0.26 or >1.65), as well as controls with a positive ATTR-CA bone scan (≥2 Perugini score).^8^

This study complies with the principles of the Declaration of Helsinki and was approved by a national ethics committee in March 2019 (CPP Ouest, n° 2018-004054-24). The study protocol was released on the ClinicalTrials.gov site under the number NCT03616496.

### PET/Computed Tomography recording and reconstruction

[^18^F]-florbetaben (Neuraceq, Life Molecular Imaging, Berlin, Germany) was delivered to each participating center by Curium Pharma (Paris, France) in unit doses ready for injection. PET/computed tomography (CT) scans were recorded on a Vereos camera (Philips Healthcare) in Nancy, a Biograph Vision 450 camera (Siemens Healthineers) in Paris-Henri Mondor (Creteil), a Biograph 6 or a Biograph Vision 600 (Siemens Healthineers) in Toulouse, a Biograph mCT (Siemens Healthineers) or a Discovery MI (GE Healthcare) in Rennes, and a Discovery 710 (GE Healthcare) in Nimes.

**Figure 1:**
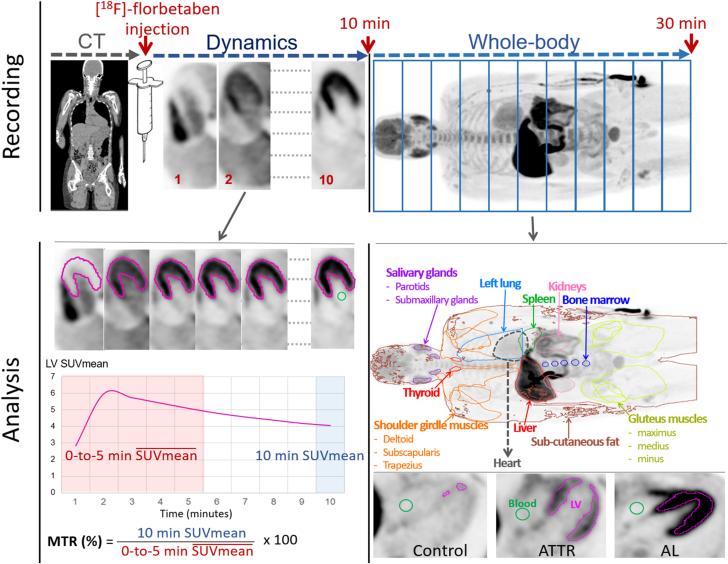
**Representation of the PET/CT Recording and Image Analysis Procedures** AL = light chain amyloidosis; ATTR = transthyretin amyloidosis; LV = left ventricular; MTR = myocardial tracer retention index; PET = positron emission tomography; SUV = standardized uptake value.

As illustrated in Figure 1, a whole-body CT scan was recorded from the skull to the mid-thigh. Thereafter, a 10-minute dynamic (1 minute per frame) single-bed position PET was recorded over the heart, starting just before the slow (30-second) intravenous injection through a short infusion tubing (≤40 cm) of 4 MBq/kg of [^18^F]-florbetaben, followed by a 10 mL saline flush. The whole-body PET scan was initiated immediately after this dynamic recording, covering the entire body from the skull to the mid-thigh, with a recording time per bed position adjusted to achieve a total of 20 minutes. This allowed the total PET recording to be completed within the 30 minutes following the [^18^F]-florbetaben injection. PET images were corrected for scatter, attenuation, accidental coincidences, radioactive decay, and planned to be reconstructed with parameters selected from a recommended methodology for harmonizing the performance of PET cameras^9^ (refer to Supplemental Table 1 for more details).

### Quantitative image analysis

All image analyses were conducted at the Nancy Core Laboratory. Myocardial uptake areas were delineated on PET images with a previously described method.^10^^,^^11^ The LV myocardial uptake volumes (MUV) were delimited on the whole-body PET images, as well as on one of the 2 last 1-minute dynamic PET images, provided that the activity identified clearly exceeded blood activity—that is, areas with standardized uptake value (SUV) >150% of blood atrial SUVmax (Figure 1). Where necessary, the epicardial fat, liver, atrial, and right ventricular walls were erased manually from the MUV. The atrial blood activity was measured from spherical volumes of interest (VOI) with diameters of 1.9 to 2.1 cm placed at the center of the right atrium on whole-body PET images and the left atrium on one of the last dynamic PET images (Figure 1). The atrial VOI and MUV limits, which were determined on one of the last dynamic PET images, were pasted unchanged on the other dynamic PET images.

An LV myocardial tracer retention index was determined using a similar method from the literature,^5^ with the LV myocardial SUVmean from the last dynamic PET image expressed as a percentage of the average LV myocardial SUVmean from initial 5-minute dynamic PET images (Figure 1).

Extracardiac organs considered are detailed in Figure 1. Their mean SUVs were determined using fully automated organ segmentation of the CT slices provided by the Total Segmentator deep learning model.^12^ This segmentation was pasted unaltered onto the corresponding whole-body PET image, except in the case of 1) bone marrow, for which medullary spherical VOIs were additionally positioned within each lumbar vertebra, and 2) lung areas which totally excluded the right lung, confounded by the interference of significant liver activity.

### Statistical analysis

Categorical variables are expressed as numbers (percentages), and continuous variables as median (IQR). The 3-group unpaired comparisons of quantitative variables were performed using the Kruskal-Wallis test, and categorical variables were analyzed using Fisher exact test. The Benjamini-Hochberg method was used to adjust the *P* values for multiple comparisons and to mitigate false-discovery rates. When significant, post hoc pairwise comparisons were performed using Dunn and Fisher exact tests for quantitative and categorical variables, respectively.

To predict groups with PET parameters, multivariate analyses were performed using bias-reduced multinomial regression (brglm2 R package) and AL-CA as the reference group. An exhaustive search minimizing the Bayesian Information Criterion was done to find the best variable combination (glmulti R package) among those with a *P* < 0.05 at univariate analysis. For all tests, *P* < 0.05 were considered significant. Optimal thresholds were determined with the closer-to-corner approach on receiver operating curves. Agreement between PET-based classification and the reference diagnostic groups (control, ATTR-CA, and AL-CA) was assessed using Cohen’s kappa coefficient. Interobserver agreement between 2 independent readers was also evaluated using Cohen’s kappa coefficient for the PET-based categorical classification. Statistical analyses were performed using IBM SPSS Statistics (Version 25) and R software version 4.1.1 (R Foundation for Statistical Computing).

## Results

### Main characteristics of the study groups

The analysis finally included a total of 61 patients (11 controls, 25 ATTR-CA, and 25 AL-CA patients). AL-CA was diagnosed through cardiac biopsies in 7 patients and extracardiac biopsies in the remaining 18. For ATTR-CA, the diagnosis was always achieved through the combination of a positive bone scintigraphy (Perugini score ≥2) and the absence of any monoclonal gammopathy with abnormal free light-chain ratio in plasma.^8^ One patient had an additional ATTR diagnosis through cardiac biopsy.

No biopsy was performed in the control patients but all underwent a bone scan and a plasma assay, which were negative (Perugini score <2 and no significant monoclonal gammopathy with abnormal free light-chain ratio).

As detailed in Table 1, ATTR-CA patients had comparable characteristics to the control group, except that they exhibited a lower body mass index and a higher septal thickness. AL-CA patients had more distinct features, when compared to the 2 other groups, they were younger, had lower rates of coronary artery disease history, and fewer specific cardiovascular risk factors. A tafamidis treatment had been initiated in 28% (7/25) of ATTR-CA patients at the time of the PET investigation, and for a median of 49 (28-206) days. A daratumumab treatment had been started in 64% (16/25) of AL-CA patients, and for a median of 43 (17-52) days. In addition, 38% (8/25) of AL-CA patients had an implanted cardiac defibrillator.

**Table 1:** Main Baseline Characteristics of Patients in the Control, ATTR-CA, and AL-CA Groups, With *P* Values for Intergroup Comparisons for Variables With Adjusted Overall *P* < 0.05

				*P* Values		
	Controls (n = 11)	ATTR-CA (n = 25)	AL-CA (n = 25)	Controls vs ATTR	Controls vs AL	ATTR vs AL
Age (y)	78.4 [72.6–79.1]	79.2 [73.2–84.8]	79 [75.0–85.0]	1.0000	0.0078	0.0000
Female	1 (9%)	2 (8%)	10 (40%)	1.0000	0.3480	0.0543
Height (m)	1.72 [1.68–1.77]	1.72 [1.66–1.76]	1.72 [1.63–1.79]	—	—	—
Body weight (kg)	85.0 [81.0–105.5]	75.0 [69.5–80.0]	67.0 [61.0–81.0]	0.0030	0.0001	0.8191
BMI (kg/m^2^)	29.8 [27.4-33.7]	25.4 [23.8-27.3]	24 [22.0-25.0]	0.0048	0.0000	0.2974
NYHA class 0-1	6 (54%)	7 (28%)	6 (24%)	—	—	—
2	4 (36%)	8 (32%)	14 (56%)			
3-4	1 (9%)	10 (40%)	5 (20%)			
Septal thickness (mm)	14 [13–14]	17 [15–20]	15 [14–17]	<0.001	0.175	0.050
Perugini score 0	11 (100%)	0 (0%)	18 (72.0%)			
1	0 (0%)	0 (0%)	5 (20.0%)			
2	0 (0%)	8 (32.0%)	2 (8.0%)			
3	0 (0%)	17 (68.0%)	0 (0%)	<0.001	1.000	<0.001
CAD history	4 (36.4%)	8 (32.0%)	0 (0%)	1.000	0.017	0.012
ICD	0 (0%)	0 (0%)	8 (32.0%)	1.000	0.227	0.012
Pacemaker	2 (18.2%)	5 (20.0%)	0 (0%)	—	—	—
Hypertension	10 (90.9%)	16 (64.0%)	9 (36%)	0.381	0.010	0.266
Diabetes	3 (27.3%)	7 (28.0%)	5 (20.0%)	—	—	—
Dyslipidemia	9 (81.8%)	16 (64%)	3 (12%)	1.000	<0.001	0.001
Tafamidis	0 (0%)	7 (28%)	0 (0%)	0.228	1.000	0.030
Daratumumab	0 (0%)	0 (0%)	16 (64%)	1.000	0.002	<0.001

AL = light chain amyloidosis; ATTR = transthyretin amyloidosis; BMI = body mass index; CA = cardiac amyloidosis; CAD = coronary artery disease; ICD = implantable cardioverter-defibrillator.

The radiation dose per patient, associated with the injection of [^18^F]-florbetaben, was 5.7 (4.9-6.7) mSv.

### Cardiac PET predictors

**Figure 2:**
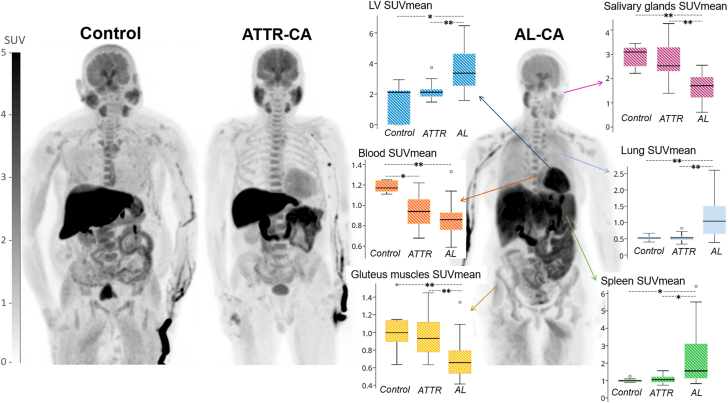
**Representative PET Images With 3-Group Comparisons of SUVmean Predictors** Whole-body PET images are displayed as maximal intensity projection (MIP) images in control, ATTR-CA, and AL-CA patients, and the 3-group comparisons are shown as box plots. ∗∗*P* < 0.001 and ∗*P* < 0.01. CA = cardiac amyloidosis; other abbreviations as in Figure 1.

**Figure 3:**
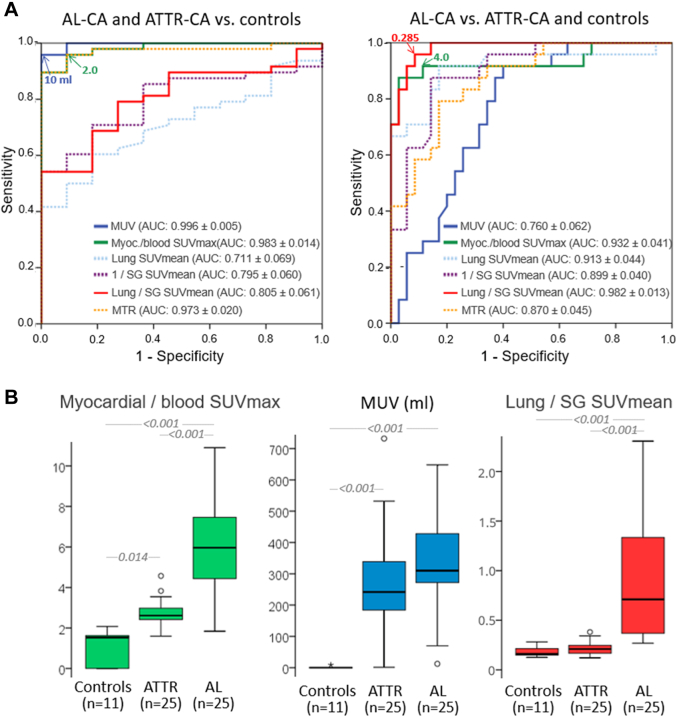
Patient Group Differentiation Using ROC Curves and Box Plots The ROC curves are presented in the upper section (A) to differentiate the AL-CA group and the ATTR-CA group from the control group (on the left), and the AL-CA group from the combination of the ATTR-CA and control groups (on the right) based on various PET parameters. The box plots shown in the lower section (B) compare the myocardial uptake volume (MUV), the myocardium/blood SUVmax ratio, and the lung/salivary gland (SG) SUVmean among the control group, ATTR-CA patients, and AL-CA patients. AUC = area under curve; ROC = receiver operating characteristic; other abbreviations as in Figures 1 and 2.

As detailed in Table 2 and illustrated in Figures 2 and 3, all PET parameters extracted from the cardiac area were significant univariate predictors of the 3-group classification, including the myocardial tracer retention index extracted from dynamic PET images, and several whole-body PET parameters—that is, MUV, myocardial and blood SUVmax and SUVmean, and myocardium/blood SUVmax and SUVmean ratios. However, by a multivariate analysis where all the Table 2 variables were entered, the only selected cardiac PET predictor of AL-CA and ATTR-CA was the myocardium/blood SUVmax ratio extracted from whole-body PET images (Table 3).

**Table 2:** Median Values (IQRs) Obtained in Control, ATTR-CA, and AL-CA Patients for PET Variables Extracted From the Cardiac Areas During Early Dynamic PET (MTR) and Whole-Body PET (MUV and SUV Variables), and With *P* Values for Intergroup Comparisons

	Controls (n = 11)	ATTR-CA (n = 25)	AL-CA (n = 25)	*P* Values		
				Controls vs ATTR	Controls vs AL	ATTR vs AL
Early dynamic PET						
MTR (%)	45.2 [42.5–52.4]	69.7 [63.1–83.1]	86.1 [80.7–103.1]	0.004	<0.001	0.005
Whole-body PET						
Myocardium						
MUV (mL)	0.08 [0.00–0.50]	242 [181–356]	310 [269–438]	<0.001	<0.001	0.322
SUVmean	2.12 [0.00–2.27]	2.12 [1.82–2.35]	3.37 [2.54–4.78]	0.080	0.001	<0.001
SUVmax	2.16 [0.00-2.78]	3.00 [2.66-3.57]	5.41 [3.74-9.54]	1.000	<0.001	<0.001
Blood						
SUVmean	1.17 [1.11–1.25]	0.94 [0.81–1.08]	0.86 [0.75–0.94]	0.003	<0.001	0.599
SUVmax	1.41 [1.30–1.66]	1.13 [1.02–1.40]	1.01 [0.92–1.19]	0.019	<0.001	0.227
Myocardium/blood						
SUVmean	1.82 [0.00–1.96]	2.30 [2.03–2.37]	4.42 [3.00–5.57]	0.014	<0.001	<0.001
SUVmax	1.53 [0.00–1.65]	2.61 [2.38–2.98]	5.96 [4.44–8.00]	0.008	<0.001	<0.001

MTR = myocardial tracer retention index; MUV = myocardial uptake volume; PET = positron emission tomography; SUV = standardized uptake value; other abbreviations as in Table 1.

**Table 3:** ORs With Corresponding 95% CIs for the Multivariate Predictors of AL-CA and ATTR-CA Patients, Which had Been Selected Among Only Cardiac, Both Cardiac, and Extracardiac PET Variables, and With the Addition of the Perugini Score Reported From the Bone Scan

	ATTR-CA Prediction			AL-CA Prediction		
	OR	95% CI	*P* values	OR	95% CI	*P* values
Only cardiac PET predictors						
Myocardium/blood SUVmax	131.73	(2.92–5934.61)	0.012	569.00	(11.45–28,274.11)	0.001
Cardiac with extracardiac PET predictors						
MUV (mL)	1.06	(1.02-1.10)	0.003	1.06	(1.02–1.10)	0.003
Lung SUVmean	1.6 × 10^-11^	(1.71 × 10^-19^; 1.50 × 10^-3^)	0.008	1.22 × 10^-2^	(4.79 × 10^-4^; 0.312)	0.008
Salivary glands SUVmean	2.07 × 10^-3^	(9.56 × 10^-6^; 4.51 × 10^-1^)	0.024	4.14 × 10^-5^	(4.06 × 10^-8^; 4.21 × 10^-2^)	0.004
Cardiac with extracardiac PET predictors and the bone scan Perugini score						
Myocardium/blood SUVmax	1.87	(0.46-7.64)	0.381	4.86	(1.48-16.00)	0.009
Perugini score	28.6	(2.32-352.96)	0.009	1.74	(0.21-14.70)	0.611

Abbreviations as in Tables 1 and 2.

This ratio gradually increased between control, ATTR-CA, and AL-CA groups; with optimal thresholds of 2 and 4 myocardium/blood SUVmax ratios, respectively, selected to differentiate the 2 CA groups from controls and the AL-CA group from the other 2 groups (Figure 3).

These thresholds correctly identified 90% (55/61) of patients as controls, ATTR-CA, or AL-CA, with a Cohen’s kappa value of 0.819 (95% CI: 0.694-0.944).

In addition, the interobserver reproducibility of this SUVmax ratio-based classification (ie, <2, 2 to −4, and >4 SUVmax ratio) was tested between 2 physicians (P.Y.M., T.S.) and showed 95% agreement (58/61, Cohen’s kappa value: 0.847 [95% CI: 0.722-0.972]).

### Extracardiac with cardiac whole-body PET predictors

As detailed in Table 4 and illustrated in Figure 2, increased SUVmean was observed in CA and mainly in AL-CA, when compared to controls, for the lung and spleen, in addition to LV myocardium, whereas decreased SUVmean was observed for blood, salivary glands, skeletal muscle, and liver.

**Table 4:** Median Values (IQRs) Obtained in Control, ATTR-CA, and AL-CA Patients for Whole-Body SUVmean Extracted Outside the Cardiac Area, and With *P* Values for Intergroup Comparisons for Variables With Adjusted Overall *P* < 0.05

	Controls (n = 11)	ATTR-CA (n = 25)	AL-CA (n = 25)	*P* Value		
				Controls vs ATTR	Controls vs AL	ATTR vs AL
Salivary glands	3.09 [2.49–3.37]	2.52 [2.30–3.29]	1.70 [1.21–2.07]	1.0000	0.0000	0.0000
Thyroid	1.15 [1.07–1.22]	1.01 [0.95–1.19]	1.12 [1.02–1.48]	—	—	—
Left lung	0.52 [0.48–0.56]	0.53 [0.46–0.57]	1.04 [0.65–1.51]	1.0000	0.0002	0.0000
Liver	8.30 [7.42–9.59]	7.55 [6.47–9.20]	6.45 [5.16–7.19]	0.5026	0.0021	0.0309
Spleen	0.99 [0.91–1.05]	1.05 [0.90–1.22]	1.55 [1.12–3.12]	1.0000	0.0025	0.0013
Kidneys	2.19 [1.90–2.48]	1.82 [1.62–2.25]	1.87 [1.67–2.23]	—	—	—
Bone marrow	3.48 [2.87–4.23]	2.82 [2.41–3.12]	3.23 [2.82–4.17]	0.0286	1.0000	0.0347
Gluteus muscles	1.00 [0.83–1.15]	0.93 [0.78–1.12]	0.66 [0.54–0.79]	1.0000	0.0009	0.0008
Shoulder muscles	0.80 [0.72–0.93]	0.72 [0.68–0.93]	0.63 [0.58–0.70]	1.0000	0.0133	0.0109
Subcutaneous fat	0.38 [0.36–0.42]	0.42 [0.38–0.48]	0.38 [0.37–0.52]	—	—	—

Abbreviations as in Tables 1 and 2.

Among all the cardiac and extracardiac univariate predictors from ^18^F-florbetaben PET (ie, all Table 2 variables and variables for which pairwise comparison *P* values are indicated in Table 4), the multivariate predictors of AL-CA or ATTR-CA were MUV, lung SUVmean, and salivary gland SUVmean (Table 3).

As detailed in Figure 3, a larger MUV allowed differentiation of the 2 CA groups from controls with an optimal threshold of 10 mL. In contrast, higher lung SUVmean and lower salivary gland SUVmean were predominantly able to differentiate AL-CA from the 2 other groups (Figure 2).

To further simplify, these latter 2 predictors were combined in a simple ratio (ie, the lung/salivary gland SUVmean ratio). As shown in Figure 3, this ratio resulted in a large area under the receiver operating curve of 0.957 ± 0.024 for distinguishing the AL-CA group from the other 2 groups. An optimal threshold of 0.285 was subsequently selected to differentiate AL-CA from the other 2 groups.

The criterion of a lung/salivary gland SUVmean >0.285 for AL-CA, and the criteria of a >10 mL MUV but a <0.285 lung/salivary gland SUVmean ratio for ATTR-CA allowed to correctly identify 93% (57/61) of patients as either controls, ATTR-CA, or AL-CA. The Cohen’s kappa value for this prediction was 0.871 (95% CI: 0.761-0.981).

As also detailed in Table 3, the PET myocardium/blood SUVmax ratio remained a significant multivariate predictor of the 3-group classification, when the bone scan Perugini score was added to the analysis.

Representative examples of whole-body PET images from each group are shown in Figure 2 and other ones in the Central Illustration, and the 3-group comparisons of the CT density and volume of the considered extracardiac organs are detailed in Supplemental Table 2.

**Central Illustration:**
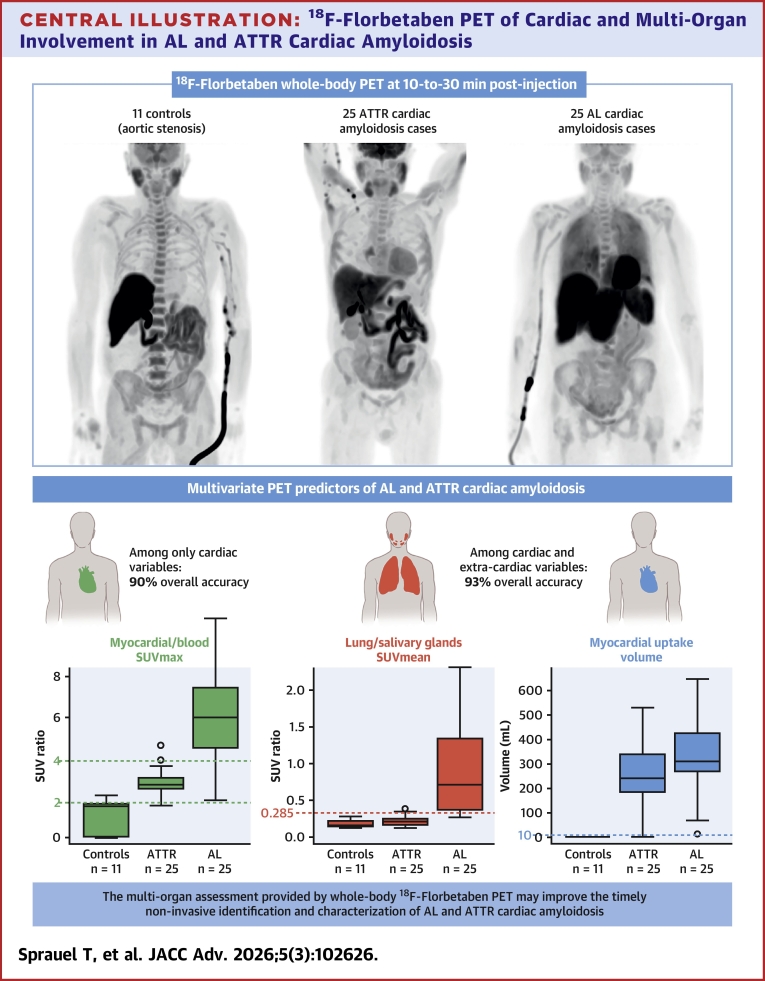
^18^F-Florbetaben PET of Cardiac and Multi-Organ Involvement in AL and ATTR Cardiac Amyloidosis Representative whole-body PET images are displayed as maximal intensity projection (MIP) images in control, ATTR-CA, and AL-CA patients (upper half), and results of the multivariate 3-group predictions are shown as box plots (lower half). Abbreviations as in Figures 1 and 2.

The STROBE (Strengthening The Reporting Of Observational Studies in Epidemiology) checklist has been added as a Supplemental Appendix.

## Discussion

This prospective multicentric study shows that a whole-body ^18^F-florbetaben PET allows to identify amyloidosis, as well as to differentiate between its 2 main forms, namely AL-CA and ATTR-CA, using the myocardium/blood SUVmax ratio, with this identification further strengthened by assessing cardiac involvement along with the common multi-organ involvement of AL-CA patients.^13^

Previous studies had already shown that ^18^F-florbetaben PET provided a means of detecting CA.^3^, ^4^, ^5^ However, this detection, predominantly based on dynamic pharmacokinetic analyses, was less efficient for ATTR-CA than for AL-CA, likely due to differences in tracer affinity.^3^, ^4^, ^5^ These dynamic pharmacokinetic analyses are often challenging as this tracer is highly lipophilic and sticky (see the residual activities in perfusion tubes and brachial veins in Figure 2). We therefore hypothesized that CA could be identified and characterized with a more conventional whole-body ^18^F-florbetaben PET scan and analysis of both cardiac and extracardiac involvement sites.

In the present study, the uptake of cardiac ^18^F-florbetaben was quantified using a method previously developed for detecting myocarditis from PET images.^10^^,^^11^ This method is based on the principle that an LV uptake exceeding 150% of the maximum blood activity (auricular blood SUVmax) is highly indicative of a myocardial origin. This approach helps to prevent the mixing of myocardial and blood activities caused by cardiac motion and partial volume effects.

Our results were remarkably similar to those previously observed in myocarditis^10^^,^^11^ with efficient CA detection provided by the MUV, and the myocardium/blood SUVmax ratio (Figure 3). However, in contrast to MUV, the SUVmax ratio was able to further differentiate between AL-CA and ATTR-CA (Figure 3), most likely due to greater affinity of the tracer for AL fibrils.

Consequently, the myocardium/blood SUVmax ratio provided an accurate detection of CA and characterization of CA forms, with overall accuracies of 90% for identifying ATTR-CA, AL-CA with the respective criteria of 2 to 4, and >4 SUVmax ratios. This SUVmax ratio-based prediction outperformed that provided by a myocardial retention index that was computed from dynamic postinjection PET images.

The characterization of CA forms was further strengthened by additional consideration of ^18^F- florbetaben uptake outside of the heart region. While ATTR-CA mainly affects the heart, AL-CA is indeed known to commonly present with multi-organ involvement.^13^

Compared to the AL-ATTR and control groups, our AL-CA patients exhibited significant increases in ^18^F-florbetaben uptake within the lung and spleen (Figure 2). This observation is consistent with previous autopsy and transbronchial biopsy studies, which revealed alveolar–septal wall amyloid deposition in most AL patients.^14^ In contrast, such findings were less common in ATTR patients.^14^^,^^15^ Furthermore, [^18^F]florbetapir, another amyloid PET tracer, had already been shown to identify pulmonary AL amyloidosis.^16^

Splenic amyloid deposition is also common in AL but rarely occurs in ATTR amyloidosis, as previously established by studies with ^123^I-labeled serum amyloid P component scintigraphy,^17^ HMDP scintigraphy,^18^ pathology examinations,^19^ and MRI.^20^ Specifically, the spleen/skeletal muscle signal ratios determined by short tau inversion recovery or late gadolinium enhancement MRI sequences were previously found to identify AL-CA patients.^20^ In the present study, the spleen/gluteus muscle SUVmean ratio was also a strong predictor of AL-CA, slightly below the lung/salivary gland SUVmean ratio (results not shown).

Taken together, these findings highlight the effectiveness of identifying AL-CA by ratios between organs with elevated image signals, such as the myocardium, lung, or spleen, and organs where the signal remains unchanged or decreases, such as blood, salivary glands, or skeletal muscle. In addition, for ^18^F-florbetaben PET, SUV ratios could be less affected by the varying amounts of tracer that remain in perfusion tubes and brachial veins than absolute SUV values (Figure 2).

The reasons why several organs and tissues of CA patients exhibited decreased ^18^F-florbetaben uptake, compared with controls, warrant further investigation. Increased initial uptake of ^18^F-florbetaben in amyloid organs may explain why residual tracer activity was lower in the blood of CA patients, as well as the liver (Table 4), given that the liver plays a crucial role in the clearance of ^18^F-florbetaben from the blood.

Decreased ^18^F-florbetaben uptake was also observed in skeletal muscle as well as the salivary glands of AL-CA patients (Table 4, Figure 2). Considering that fatty organs show nonspecific uptake of lipophilic amyloid PET tracers,^21^ the decreased uptake in the salivary glands may be partly explained by organ atrophy with fat wasting, analogous to that observed in organs of cancer patients.^22^ In a recent study, the ^18^F-florbetaben uptake of salivary glands has been considered as a fat-related marker.^23^ Moreover, our CT scan data, detailed in Supplemental Table 2, indicate that the salivary glands of AL-CA patients had reduced volumes and signs of decreased fat content, evidenced by increased CT densities. These observations may also extend to skeletal muscle, although only nonsignificant trends were observed in terms of decreased volume and increased density in AL-CA patients.

The role of tissue atrophies due to: 1) an AL amyloid neuropathy^24^; 2) damage to tissue in contact with amyloid fibrils^25^; or 3) a nonspecific manifestation of the cachexia affecting severely ill patients, which also warrants further discussion. Nevertheless, from a diagnostic standpoint, our study indicates that: 1) a decrease in ^18^F-florbetaben uptake in the salivary glands suggests systemic involvement related to AL-CA; and 2) the predictive accuracy for AL-CA is further strengthened when the ratio of lung uptake over salivary gland uptake is used (Figure 3).

The lung over salivary gland uptake ratio was a main multivariate predictor of the 3-group classification. MUV was a secondary predictor that differentiated AL-CA and ATTR-CA patients from controls (Figure 3). Using these 2 predictors, the overall accuracy for identifying ATTR-CA and AL-CA reached 93%. This accuracy was slightly but not significantly higher than the 90% achieved with the myocardial/blood SUVmax ratio.

The myocardial/blood SUVmax ratio further enhanced the predictive ability of the bone scan Perugini score for identifying the 3 groups, consistent with the limited ability of the Perugini score to separate AL-CA from control patients (Table 3). In addition, the imperfect specificity of the Perugini score is illustrated by the fact that it was > 0 in several patients with biopsy-proven AL amyloidosis. Notably, 2 of these patients had a Perugini score of 2 (Table 1).

It is important to emphasize that ^18^F-florbetaben uptake was assessed in the lung, salivary glands, and other extracardiac organs using the fully automated segmentation process from the freely available TotalSegmentator deep learning model.^12^ This approach guarantees the consistency between different observers. This is in contrast to the cardiac analysis method which requires manual adjustments for positioning the blood auricular VOI and for excluding nonmyocardial uptake areas. The latter method nevertheless demonstrated high interobserver reproducibility, achieving a 95% agreement rate for identifying patients with myocardium/blood SUVmax ratios evocative of AL-CA or ATTR-CA (>4 and 2 to −4 SUVmax ratio, respectively).

### Study Limitations

The main limitations of the study are its small sample size, the lack of prospective validation of the PET-based diagnostic thresholds, and the restriction of the cohort to 1) wild-type ATTR-CA cases and 2) patients already diagnosed with either CA-AL or ATTR-AL. Study patients were therefore not in phase with the standard diagnostic workflow. Consequently, further studies involving larger populations and patients with suspected CA are necessary. Further research should also involve patients in the “grey zone” of equivocal bone scintigraphy and/or coexisting monoclonal gammopathy, where alternatives to biopsy are still lacking.

Cardiac hypertrophy could be attributed to a nonamyloidosis cause with a low margin of error in our control group of patients with aortic stenosis who required transcatheter aortic valve implantation or surgical treatment. The absence of any amyloidosis sign on bone scans or plasma tests further strengthened this nonamyloidosis origin. However, our results need to be confirmed in populations with cardiac hypertrophy caused by factors other than aortic stenosis or amyloidosis, such as hypertensive heart disease or hypertrophic cardiomyopathies.

## Conclusions

The present prospective multicentric study demonstrates that a whole-body ^18^F-florbetaben PET may identify and characterize AL-CA and ATTR-CA, based on the myocardium/blood SUVmax ratio. This identification may be further strengthened by the whole-body assessment of AL-related multi-organ involvement, specifically the lung and salivary glands.

Perspectives **COMPETENCY IN MEDICAL KNOWLEDGE:** The multi-organ assessment provided by whole-body ^18^F-florbetaben PET may improve the timely noninvasive identification and characterization of AL-CA and ATTR-CA.

**TRANSLATIONAL OUTLOOK:** This imaging technique could also be potentially beneficial for monitoring CA across multiple organs during treatment, an opportunity to develop a new research direction.

## Funding support and author disclosures

This study was funded by a French national grant (Projet Hospitalier de Recherche Clinique National [PHRC-N 2017, NCT03616496]), and ^18^F-florbetaben was provided free of charge by Curium Pharma (Paris, France). Dr Imbert has received consulting fees and research grants from 10.13039/100020587Spectrum Dynamics. Dr Verger has received consulting fees from 10.13039/100004336Novartis, Telix, GE, Curium, and Eisaï. Dr Huttin has received consulting fees from 10.13039/100006400Alnylam, 10.13039/100002491BMS, 10.13039/100004319Pfizer, and General Electric HEalthcare. Dr Donal has received consulting fees and research facilities from 10.13039/100000046Abbott, 10.13039/100004319Pfizer, 10.13039/100006400Alnylam, and General Electric HEalthcare. Dr Lairez has received speaking and consulting fees from 10.13039/100006400Alnylam, 10.13039/100015362Amicus, 10.13039/100004325AstraZeneca, 10.13039/100004326Bayer, 10.13039/100002491BMS, 10.13039/100004319Pfizer, Sanofi-Genzyme, and 10.13039/501100011699Siemens Healthineers. Dr Damy has received consulting fees and/or research grants from 10.13039/100006396Alexion, 10.13039/100006400Alnylam, BridgeBio, 10.13039/100004319Pfizer, Neurimmune, 10.13039/100004326Bayer, 10.13039/100004325AstraZeneca, 10.13039/100004336Novartis, 10.13039/501100004191Novo Nordisk, and 10.13039/100015364Prothena. Dr Marie has received consulting fees and research grants from 10.13039/100020587Spectrum Dynamics. All other authors have reported that they have no relationships relevant to the contents of this paper to disclose.
