# Supplementary material for: 18F-Florbetaben PET Detection of Cardiac and Multi-Organ Involvement for Identifying AL and ATTR Cardiac Amyloidosis
Source: JACC Adv. 2026 Mar 25;5(3):102626. doi: 10.1016/j.jacadv.2026.102626 (PMC13352038; doi:10.1016/j.jacadv.2026.102626)
Supplement: Supplemental Material [file mmc1.pdf]

Supplemental Table 1: Parameters recommended for the image reconstruction of each PET camera. These parameters were established following a methodology aimed at harmonizing the performance of PET cameras[9], and using images of an NEMA NU2-2007 body phantom filled conforming to EANM/EARL guidelines for ^{18}F–FDG Image Quality QC phantom imaging. Note that corrections for attenuation, diffusion, random coincidences, and radioactive decay were also applied.

FWHM: full width at half-maximum.

	**Nancy**	**Creteil Henri-Mondor**	**Toulouse**		**Rennes**		**Nîmes**
	Vereos (Philips)	*Biograph Vision 450 (Siemens Healthineers)*	*Biograph 6 (Siemens Healthineers)*	*Biograph Vision 600 (Siemens Healthineers)*	*Biograph mCT (Siemens Healthineers)*	*Discovery MI (GE Healthcare)*	*Discovery 710 (GE Healthcare)*
Algorithm	Iterative 3D	Iterative 3D	Iterative 2D	Iterative 3D	Iterative 3D	Iterative 3D	Iterative 3D
Iterations	2	4	3	4	2	3	2
Subsets	10	5	8	5	21	16	24
Time-of-flight	yes	yes	no	yes	yes	yes	yes
Point Spread Function	no	yes	no	yes	no	no	no
Post filter (FWHM)	Gaussian (6 mm)	Gaussian (8 mm)	Gaussian (5 mm)	Gaussian (8 mm)	Gaussian (6 mm)	Gaussian (6 mm)	Gaussian (6.4 mm)
Pixel size (mm²)	2.0x2.0	1.65x1.65	4.07x4.07	1.65x1.65	4.07x4.07	2.73x2.73	2.73x2.73
Slice thickness (mm)	2.00	1.65	2.03	1.65	2.03	2.8	3.3

Supplemental Table 2: Median values [interquartile ranges] obtained in control, ATTR- and AL-CA patients for whole-body CT variable, and with p values for intergroup comparisons for variables with adjusted overall p < 0.05.

		**Controls** [n=11]	**ATTR-CA** [n=25]	**AL-CA** [n=25]	**P values**		
					Controls vs. ATTR	Controls vs. AL	ATTR vs. AL
Salivary glands	Volume [ml]	54 [38;59]	30 [28;38]	23 [18;31]	0.0396	0.0001	0.0771
	Density [HU]	-7 [-11;8]	10 [-4;22]	23 [16;33]	0.1040	0.0003	0.0756
Thyroid	Volume [ml]	17.4 [11.6;19.4]	11.9 [10.2;15.5]	11.5 [9.8;13.9]	-------	-------	-------
	Density [HU]	60 [47;70]	65 [59;80]	66 [54;76]	-------	-------	-------
Left lung	Volume [l]	1.31 [1.19;1.85]	1.35 [1.25;1.61]	1.37 [1.09;1.56]	-------	-------	-------
	Density [HU]	-702 [-744;- 674]	-677 [-713;-651]	-659 [-725;-606]	-------	-------	-------
Liver	Volume [l]	1.68 [1.63;1.78]	1.45 [1.28;1.65]	1.65 [1.44;1.89]	-------	-------	-------
	Density [HU]	49 [45;56]	52 [48;56]	54 [47;56]	-------	-------	-------
Spleen	Volume [ml]	174 [142;235]	187 [140;235]	195 [162;251]	-------	-------	-------
	Density [HU]	33 [30;36]	37 [31;41]	33 [30;36]	-------	-------	-------
Kidneys	Volume [l]	327 [283;396]	267 [233;299]	253 [218;294]	-------	-------	-------
	Density [HU]	0.9 [17.3;21.9]	19.2 [16.5;22.7]	20.5 [16.7;22.8]	-------	-------	-------
Bone marrow	Volume [ml]	27.9 [23.6;32.1]	24.4 [20.9;29.4]	25.6 [19.9;30.7]	-------	-------	-------
	Density	97 [69;129]	87 [65;109]	114 [101;154]	-------	-------	-------
Gluteus muscles	Volume [ml]	1.97 [1.84;2.13]	1.79 [1.61;2.06]	1.65 [1.33;2.00]	-------	-------	-------
	Density [HU]	17 [3;25]	24 [14;29]	26 [14; 32]	-------	-------	-------
Shoulders muscles	Volume [ml]	1.30 [1.23;1.42]	1.20 [1.13;1.31]	0.99 [0.75;1.38]	-------	-------	-------
	Density [HU]	32 [24;37]	37 [27;40]	37 [33; 41]	-------	-------	-------
Subcutaneous fat	Volume [ml]	21.2 [19.9;27.7]	14.0 [9.6;16.1]	10.3 [6.6;13.3]	0.0044	0.0001	0.7057
	Density [HU]	-95 [-102;-93]	-89 [-92;-81]	-80 [-89; -63]	0.0192	0.0001	0.1708

Supplemental Figure 1: Time-activity curves after ^{18}F-florbetaben injection in an ATTR patient.

Right auricular blood SUVmean and septal left ventricular (LV) SUVmean, and the myocardium-to-blood SUVmean ratio were derived from a dynamic cardiac PET recorded during the 60 minutes following injection. For the present CAPRI study, we chose the 10-to-30-minute timeframe for whole-body recording based on low residual blood activity and a high and relatively stable myocardium-to-blood activity ratio.

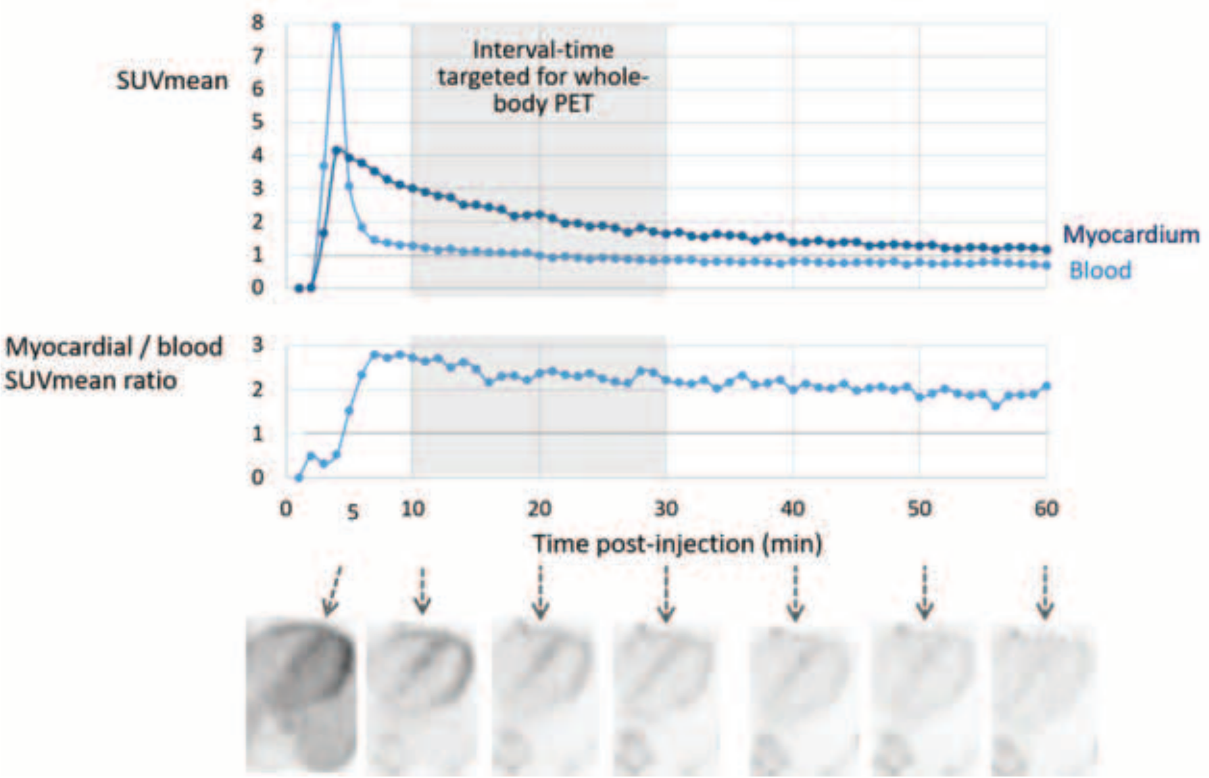

Annexe 1: STROBE Statement

	Item No.	Recommendation	Page No.	Relevant text from manuscript
**Title and abstract**	1	(*a*) Indicate the study's design with a commonly used term in the title or the abstract	p. 1	Multicentre observational cross-sectional study of ^{18}F-florbetaben PET for identifying AL and ATTR cardiac amyloidosis through cardiac and multi-organ involvement
		(*b*) Provide in the abstract an informative and balanced summary of what was done and what was found	p. 4	Background: ^{18}F-florbetaben positron emission tomography (PET) is reported to detect (i) cardiac amyloidosis (CA), particularly AL (light chain) forms, and (ii) sites of extracardiac AL-amyloid infiltrates.<br>Objectives: Identify CA and differentiate AL-CA from ATTR-CA (transthyretin) by evaluating cardiac and multi-organ involvement with whole-body 18F-florbetaben PET.<br>Methods: Multicentric study of 61 patients with left ventricular (LV) hypertrophy due to AL-CA (n=25), ATTR-CA (n=25), and aortic stenosis (n=11, controls). A 20-minute whole-body PET was preceded by a 10-minute dynamic cardiac PET recording started during 18F-florbetaben injection.<br>Results: Tracer uptake was significantly increased in whole-body PET of CA patients compared to controls, with AL-CA patients, in particular, exhibiting increased uptake in myocardium, lung, and spleen, and decreased uptake in blood, salivary glands, skeletal muscle, and liver. Among cardiac parameters, the myocardial/blood SUVmax ratio from whole-body PET best differentiated AL- CA from ATTR-CA, with 90% (55/61) of patients identified correctly (kappa value: 0.819) using SUVmax ratio thresholds of 2-to-4 for ATTR-CA and > 4 for AL-CA. When combining cardiac with extracardiac PET variables, the selected multivariate predictors were myocardial uptake volume

				(MUV), and the lung and salivary gland SUVmean. Thresholds of MUV > 10 ml for CA and lung/salivary gland SUVmean > 0.285 for AL-CA correctly identified 93% (57/61) of patients (kappa value: 0.871). PET variables improved the overall prediction provided by the bone scan Perugini score ($p < 0.001$).<br>Conclusion: Whole-body ^{18}F-florbetaben PET identifies CA and differentiates AL-CA from ATTR- CA. These distinctions are strengthened by combining assessments of cardiac and multi-organ involvement.
**Introduction**				
Background/rationale	2	Explain the scientific background and rationale for the investigation being reported	p. 6	Early and accurate diagnosis of cardiac amyloidosis has become increasingly important with the advent of disease-modifying therapies. However, non-invasive differentiation between AL and ATTR cardiac amyloidosis remains challenging, as bone scintigraphy primarily detects ATTR forms, while cardiac MRI lacks specificity. Amyloid PET tracers such as [^{18}F]-florbetaben have shown promise for detecting cardiac amyloid deposition, particularly in AL amyloidosis, and may also identify extracardiac amyloid involvement. This raises the possibility that whole-body amyloid PET could provide a comprehensive assessment of both cardiac and multi-organ amyloid burden. Based on prior pharmacokinetic studies, an early whole-body imaging window may optimize tracer uptake assessment in both AL and ATTR amyloidosis.<br>Taken together, these data suggest that whole-body [^{18}F]-florbetaben PET could provide a comprehensive assessment of cardiac and extracardiac amyloid involvement, potentially enabling improved differentiation between AL and ATTR cardiac amyloidosis.

Objectives	3	State specific objectives, including any prespecified hypotheses	p. 6	The present multicentre observational CAPRI study was designed to evaluate the ability of whole-body [^{18}F]-florbetaben PET to identify cardiac amyloidosis and to differentiate AL from ATTR forms, in comparison with a control population presenting with non-amyloid left ventricular hypertrophy.<br>We hypothesized that combining early whole-body cardiac and extracardiac PET parameters would allow accurate identification of cardiac amyloidosis and improve the discrimination between AL and ATTR subtypes.
**Methods**				
Study design	4	Present key elements of study design early in the paper	p. 7	This was a multicentre observational cross-sectional study comparing patients with AL cardiac amyloidosis, ATTR cardiac amyloidosis, and a control population with non-amyloid left ventricular hypertrophy. All participants underwent PET and all measurements were collected at a single time point.
Setting	5	Describe the setting, locations, and relevant dates, including periods of recruitment, exposure, follow-up, and data collection	p. 7	The study was conducted in five French university hospitals (Nancy, Créteil, Rennes, Toulouse, and Nîmes). The study protocol was approved by a national ethics committee in March 2019 and registered on ClinicalTrials.gov (NCT03616496). Patients were included between September 2019 and January 2025.
Participants	6	(*a*) *Cohort study*—Give the eligibility criteria, and the sources and methods of selection of participants. Describe methods of follow-up<br>*Case-control study*—Give the eligibility criteria, and the sources and methods of case ascertainment and control selection. Give the rationale for the choice of cases and controls<br>*Cross-sectional study*—Give the eligibility criteria, and the sources and methods of selection of participants	p. 7	Eligible patients were prospectively assigned to three groups: AL cardiac amyloidosis, ATTR cardiac amyloidosis, and controls with non-amyloid left ventricular hypertrophy related to aortic stenosis based on established diagnostic criteria before the PET exam.<br>Inclusion criteria for ATTR cardiac amyloidosis were a positive biopsy or a positive bone scintigraphy (Perugini score ≥ 2), absence of family history, and suspected wild-

				type ATTR. AL cardiac amyloidosis required biopsy-proven amyloid deposition associated with abnormal cardiac biomarkers. Control patients had a recent history of surgical or transcatheter aortic valve implantation.
		(*b*) *Cohort study*—For matched studies, give matching criteria and number of exposed and unexposed<br>*Case-control study*—For matched studies, give matching criteria and the number of controls per case	NA	
Variables	7	Clearly define all outcomes, exposures, predictors, potential confounders, and effect modifiers. Give diagnostic criteria, if applicable	p. 8 and 9	The primary outcomes were the identification of cardiac amyloidosis and the differentiation between AL and ATTR subtypes. PET-derived variables included myocardial and extracardiac [^{18}F]-florbetaben uptake parameters, such as myocardial SUV metrics, myocardial-to-blood activity ratios, myocardial uptake volume, myocardial tracer retention index, and organ-specific SUVmean values.
Data sources/ measurement	8*	For each variable of interest, give sources of data and details of methods of assessment (measurement). Describe comparability of assessment methods if there is more than one group	p. 8 and 9	PET/CT data were acquired using standardized whole-body and dynamic cardiac imaging protocols across participating centers. Image reconstruction was harmonized according to published recommendations. Quantitative PET analyses were centrally performed at the Nancy core laboratory using predefined methods for myocardial and blood pool measurements, while extracardiac organ uptake was assessed using automated CT-based segmentation.
Bias	9	Describe any efforts to address potential sources of bias	p. 8 and 9	To minimize measurement and inter-center variability, PET acquisition and reconstruction protocols were standardized, and all image analyses were performed centrally by a core laboratory using predefined quantitative methods. Potential spillover and segmentation biases were addressed through organ-specific exclusions and manual corrections when required.
Study size	10	Explain how the study size was arrived at	p. 8 and 9	The study was designed to include up to 25 eligible patients

	in each of the three study groups. While the target sample size was reached for the AL and ATTR cardiac amyloidosis groups, only 11 control patients could be enrolled, reflecting the limited availability of eligible controls during the study period.

Continued on next page

Quantitative variables	11	Explain how quantitative variables were handled in the analyses. If applicable, describe which groupings were chosen and why	p. 8 and 9	Quantitative PET variables were analyzed either as continuous measures or as derived parameters. Myocardial uptake was expressed using standardized uptake values and myocardial-to-blood activity ratios. Myocardial uptake volume and myocardial tracer retention index were calculated using predefined thresholds relative to blood pool activity. Extracardiac tracer uptake was assessed using organ-specific SUVmean values. When appropriate, continuous variables were further categorized using predefined thresholds to differentiate between cardiac amyloidosis subtypes.
Statistical methods	12	(*a*) Describe all statistical methods, including those used to control for confounding	p. 9 and 10	Categorical variables were expressed as numbers and percentages, and continuous variables as medians with interquartile ranges. Group comparisons were performed using the Kruskal–Wallis test for quantitative variables and Fisher’s exact test for categorical variables, with post-hoc pairwise comparisons using Dunn’s and Fisher’s exact tests, respectively. P-values were adjusted for multiple comparisons using the Benjamini–Hochberg method. Multivariate analyses were conducted using bias-reduced multinomial logistic regression to predict diagnostic groups based on PET-derived parameters, with AL cardiac amyloidosis as the reference category. Variable selection was performed using an exhaustive search minimizing the Bayesian Information Criterion. Receiver operating characteristic curve analysis with a closer-to-corner approach was used to determine optimal thresholds. Agreement between PET-based classification and the reference diagnostic groups (control, ATTR-CA, and AL-CA) was assessed using Cohen’s kappa coefficient. Inter-observer agreement between two independent readers was also evaluated using Cohen’s kappa coefficient for the PET-

				based categorical classification. Statistical analyses were performed using SPSS and R software.
		(*b*) Describe any methods used to examine subgroups and interactions	NA	
		(*c*) Explain how missing data were addressed		No imputation was performed; all analyses were conducted on complete cases.
		(*d*) *Cohort study*—If applicable, explain how loss to follow-up was addressed<br>*Case-control study*—If applicable, explain how matching of cases and controls was addressed<br>*Cross-sectional study*—If applicable, describe analytical methods taking account of sampling strategy	NA	Agreement between PET-based classification and the reference diagnostic groups (control, ATTR-CA, and AL-CA) was assessed using Cohen's kappa coefficient. Inter-observer agreement between two independent readers was also evaluated using Cohen's kappa coefficient for the PET-based categorical classification.
		(*e*) Describe any sensitivity analyses	p. 10	
**Results**				
Participants	13*	(a) Report numbers of individuals at each stage of study—eg numbers potentially eligible, examined for eligibility, confirmed eligible, included in the study, completing follow-up, and analysed	p. 10	A total of 61 patients were included in the final analysis, comprising 25 patients with ATTR cardiac amyloidosis, 25 patients with AL cardiac amyloidosis, and 11 control patients with non-amyloid left ventricular hypertrophy.
		(b) Give reasons for non-participation at each stage	NA	
		(c) Consider use of a flow diagram	NA	
Descriptive data	14*	(a) Give characteristics of study participants (eg demographic, clinical, social) and information on exposures and potential confounders	p. 10	Baseline demographic, clinical, and imaging characteristics of the three study groups are summarized in Table 1. ATTR cardiac amyloidosis patients had characteristics comparable to controls, except for lower body mass index and higher septal thickness. AL cardiac amyloidosis patients were younger and had fewer cardiovascular risk factors. Ongoing disease-specific treatments and radiation exposure are also reported.
		(b) Indicate number of participants with missing data for each variable of interest	NA	
		(c) *Cohort study*—Summarise follow-up time (eg, average and total amount)	NA	

Outcome data	15*	*Cohort study*—Report numbers of outcome events or summary measures over time	NA	
		Case-control study—Report numbers in each exposure category, or summary measures of exposure	NA	
		Cross-sectional study—Report numbers of outcome events or summary measures	p. 10 to 12	The final analysis included 61 patients: 11 controls, 25 ATTR-CA, and 25 AL-CA.<br>Summary exposure measures showed that cardiac and extracardiac PET uptake differed across groups. The myocardium/blood SUVmax ratio increased progressively from controls to ATTR-CA and AL-CA, with thresholds of <2, 2–4, and >4 distinguishing the three groups. Extracardiac SUVmean values also varied between groups, with higher lung and spleen uptake in AL-CA and lower blood, salivary gland, skeletal muscle, and liver uptake compared with controls.
Main results	16	(*a*) Give unadjusted estimates and, if applicable, confounder-adjusted estimates and their precision (eg, 95% confidence interval). Make clear which confounders were adjusted for and why they were included	p. 11 and 12	Cardiac and extracardiac PET quantitative results are summarized in Tables 2–4. All cardiac PET parameters were significant univariate predictors of diagnostic group classification. In multivariate analysis, the myocardium/blood SUVmax ratio was the only retained cardiac predictor. When combining cardiac and extracardiac parameters, myocardial uptake volume, lung SUVmean, and salivary gland SUVmean were independently associated with cardiac amyloidosis subtype, with high diagnostic agreement.
		(*b*) Report category boundaries when continuous variables were categorized	p. 11 and 12	The myocardium/blood SUVmax ratio gradually increased between control, ATTR-CA, and AL-CA groups; with optimal thresholds of 2 and 4 myocardium/blood SUVmax ratios respectively selected to differentiate the 2 CA groups from controls and the AL-CA group from the other two groups, with 90% (55/61) of patients identified correctly

			(Figure 3).<br>The criterion of a lung/salivary gland SUVmean > 0.285 for AL-CA, and the criteria of a > 10 ml MUV but a < 0.285 lung/salivary gland SUVmean ratio for ATTR-CA allowed to correctly identify 93% (57/61) of patients as either controls, ATTR-CA, or AL-CA.
	(*c*) If relevant, consider translating estimates of relative risk into absolute risk for a meaningful time period	NA	

Continued on next page

Other analyses	17	Report other analyses done—eg analyses of subgroups and interactions, and sensitivity analyses	p. 11 and 12	Inter-observer reproducibility analyses demonstrated excellent agreement for PET-based classification. Additional analyses assessed the incremental value of PET parameters over bone scintigraphy and explored extracardiac CT characteristics.
**Discussion**				
Key results	18	Summarise key results with reference to study objectives	p. 12	This study demonstrates that whole-body [^{18}F]-florbetaben PET can identify cardiac amyloidosis and differentiate AL from ATTR forms, with the myocardium-to-blood SUVmax ratio providing high diagnostic accuracy. Diagnostic performance was further improved by incorporating extracardiac uptake patterns reflecting multi-organ involvement, particularly in AL cardiac amyloidosis.
Limitations	19	Discuss limitations of the study, taking into account sources of potential bias or imprecision. Discuss both direction and magnitude of any potential bias	p. 16	The main limitations of the study include the relatively small sample size, the lack of prospective external validation of PET-derived diagnostic thresholds, and the restriction of the cohort to patients with previously established diagnoses of wild-type ATTR or AL cardiac amyloidosis. These factors limit direct extrapolation to patients undergoing initial diagnostic evaluation.
Interpretation	20	Give a cautious overall interpretation of results considering objectives, limitations, multiplicity of analyses, results from similar studies, and other relevant evidence	p. 12 to 16	The findings are consistent with previous studies showing preferential affinity of amyloid PET tracers for AL fibrils and support the added value of whole-body imaging for capturing systemic involvement. While SUV-based ratios appear robust and reproducible, the underlying biological mechanisms of altered tracer distribution in extracardiac tissues warrant further investigation.
Generalisability	21	Discuss the generalisability (external validity) of the study results	p. 16	The generalisability of these results is limited by the

				selected study population, including controls with aortic stenosis-related hypertrophy and patients with established cardiac amyloidosis. Further studies are needed to confirm these findings in broader populations with unexplained left ventricular hypertrophy and in real-world diagnostic settings.
**Other information**				
Funding	22	Give the source of funding and the role of the funders for the present study and, if applicable, for the original study on which the present article is based	p. 2	This study was funded by a French national grant (Projet Hospitalier de Recherche Clinique National (PHRC-N 2017, NCT03616496), and 18F-florbetaben was provided free of charge by Curium Pharma (Paris, France).

*Give information separately for cases and controls in case-control studies and, if applicable, for exposed and unexposed groups in cohort and cross-sectional studies.

Note: An Explanation and Elaboration article discusses each checklist item and gives methodological background and published examples of transparent reporting. The STROBE checklist is best used in conjunction with this article (freely available on the Web sites of PLoS Medicine at http://www.plosmedicine.org/, Annals of Internal Medicine at http://www.annals.org/, and Epidemiology at http://www.epidem.com/). Information on the STROBE Initiative is available at www.strobe-statement.org.
